# A Structural Study of Epoxidized Natural Rubber (ENR-50) and Its Cyclic Dithiocarbonate Derivative Using NMR Spectroscopy Techniques

**DOI:** 10.3390/molecules170910974

**Published:** 2012-09-12

**Authors:** Rosniza Hamzah, Mohamad Abu Bakar, Melati Khairuddean, Issam Ahmed Mohammed, Rohana Adnan

**Affiliations:** 1 Nanoscience Research Laboratory, School of Chemical Sciences, Universiti Sains Malaysia, Penang 11800, Malaysia; 2 School of Industrial Technology, Universiti Sains Malaysia, Penang 11800, Malaysia

**Keywords:** ENR-50, 2D NMR, triad sequence, cyclic dithiocarbonate

## Abstract

A structural study of epoxidized natural rubber (ENR-50) and its cyclic dithiocarbonate derivative was carried out using NMR spectroscopy techniques. The overlapping ^1^H-NMR signals of ENR-50 at δ 1.56, 1.68–1.70, 2.06, 2.15–2.17 ppm were successfully assigned. In this work, the ^13^C-NMR chemical shift assignments of ENR-50 were consistent to the previously reported work. A cyclic dithiocarbonate derivative of ENR-50 was synthesized from the reaction of purified ENR-50 with carbon disulfide (CS_2_), in the presence of 4-dimethylaminopyridine (DMAP) as catalyst at reflux temperature. The cyclic dithiocarbonate formation involved the epoxide ring opening of the ENR-50. This was followed by insertion of the C–S moiety of CS_2_ at the oxygen attached to the quaternary carbon and methine carbon of epoxidized isoprene unit, respectively. The bands due to the C=S and C–O were clearly observed in the FTIR spectrum while the ^1^H-NMR spectrum of the derivative revealed the peak attributed to the methylene protons had split. The ^13^C-NMR spectrum of the derivative further indicates two new carbon peaks arising from the >C=S and quaternary carbon of cyclic dithiocarbonate. All other ^1^H- and ^13^C-NMR chemical shifts of the derivative remain unchanged with respect to the ENR-50.

## 1. Introduction

**Scheme 1:**
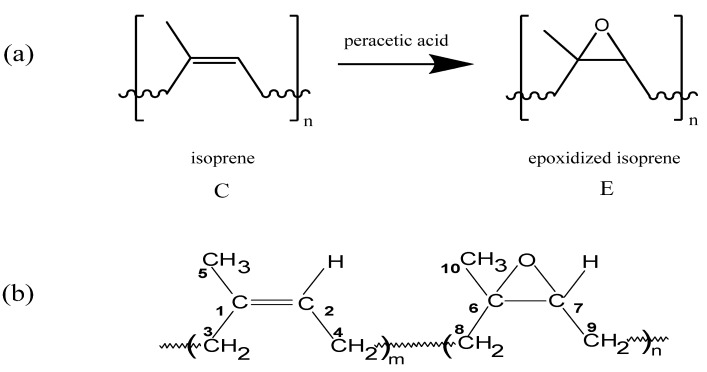
(**a**) Modification of NR to ENR [2] and (**b**) the general structure and the numbering of carbon atom in ENR-50 employed in this work.

Epoxidized natural rubber (ENR) is a modified natural rubber (NR) [1]. A typical formation of ENR from NR, *cis*-1,4-isoprene, employing peracetic acid is shown in Scheme 1a [2]. The isoprene (C) and epoxidized isoprene (E) act as monomer units that are randomly distributed along the polymer chain [1]. Various degree of epoxidation of NR is commercially available. For examples, the isoprene units in the polymer chain are 25%, 50% and 75% epoxidized in ENR-25, ENR-50 and ENR-75, respectively. For the purpose of nomenclature, the general structure and the numbering of carbon atoms in ENR-50 is shown in Scheme 1b.

NMR spectroscopy techniques are widely used to characterize various polymers either in liquid or solid state. Characterization using 1D NMR gives structural elucidation on reactive groups and monomer units in the polymer chain. However, the 2D NMR extents detail structural arrangements of the monomer units within the polymer chain. Either or both of these techniques have been applied to ENR-25 [3], ENR-50 [4,5,6,7,8,9], and ENR-75 [6], and other ENR-related compounds [10]. However, most of these works fall short of full structural assignments due to the overlapping of signals arising from the randomly distributed C and E monomer units [11,12].

**Figure 1:**
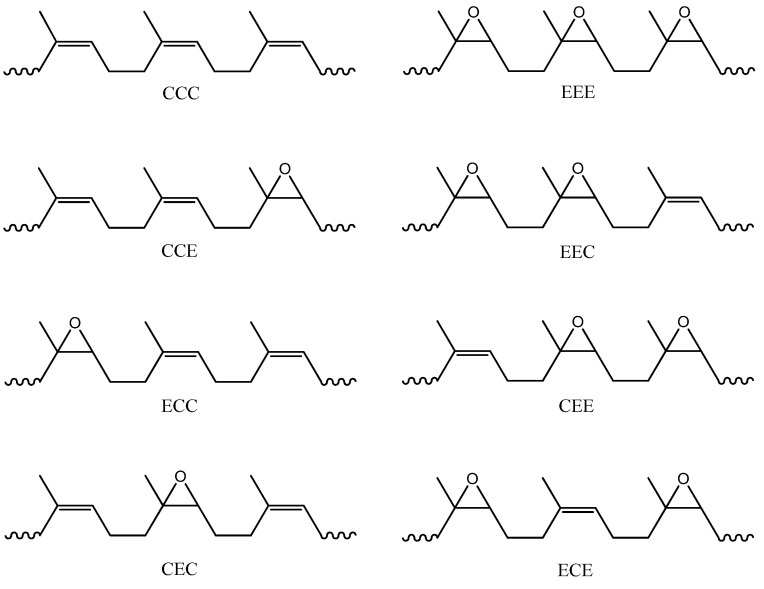
The possible triad sequence of ENR-50.

The random arrangement of C and E units within the ENR chains give rise to several probable sequences. This inevitably makes the structural interpretation via the NMR techniques difficult. To overcome this, Bradbury and Perera [12] have established a procedure of grouping the randomly located monomer units into triad sequence. A triad is made up of three possible monomer units. All possible triad sequences of ENR-50 are shown in Figure 1.

In a triad, the neighboring units may dictate the ^1^H and ^13^C environments of the middle unit. It is the middle unit that will reveal the triad structure. For example, an ENR chain fragment may comprise of C unit attached to an E unit which in turn is attached to another E unit. Thus, this triad sequence is denoted as CEE. Triad comprising of similar unit is only denoted by a single unit. Triad CCC is denoted as C and triad EEE is denoted as E. The methyl of the middle unit in the CEE and CCC triads are referred to as CE^10^E and C^5^, respectively, and the methylene carbon of C^3^ refers to the third carbon in the ENR-50 structure as shown in Scheme 1b. This also applies to the proton in the ENR-50 structure.

Thus prior to the advent of the triad sequence, previous workers [6,7,8,9,12,13] were only able to ascertain the assignments of ^1^H-NMR chemical shifts of the methine proton of C and E units but sporadically to either the methyl and or methylene protons of C and E of the ENR. Saito *et al.* [11] have reported the use of computer simulation [14,15] to predict chemical shifts of these protons and their triad assignments.

Gelling [2], on the other hand, has assigned the triad sequence based on ^13^C-NMR chemical shifts for ENR-20. However, the assignment deals with certain triad sequences such as CEE, CEC, C, EEC, and ECE and the position of carbon represented by the triad sequences was found to be inconsistent. Consequently, Saito *et al.* [11] have successfully interpreted the ^13^C-NMR spectra for the ENR related compounds and assigned their triad sequence as shown in Table 1, columns 2 and 4.

**Scheme 2:**
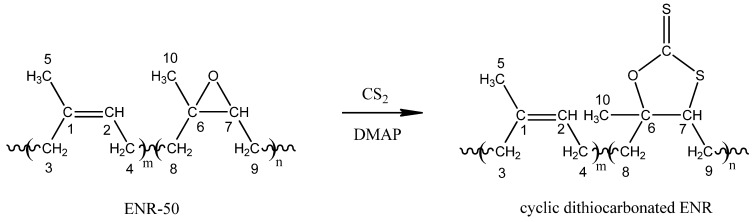
Reaction of purified ENR-50 with carbon disulfide catalyzed by 4-dimethylaminopyridine (DMAP).

The increasing interests in the science and technology of ENR in various fields such as polymer blends [16], polymer modifications [17], polymer composites [18] and advanced green materials [19,20,21] makes it inevitable to understand the detail structure of ENR. This fundamental study is important to enable comprehensive structural characterization of the desired materials. Here we attempted a complete assignment of the commercial ENR-50 using the 1D and 2D NMR spectroscopy techniques. Our work differs from other reported works [11,22,23] in term of the degree of epoxidation and physical form of rubber. The ENR used is a low molecular weight fraction of ENR-50 obtained by solvent separation [19]. In this work, the results reported by Saito *et al.* [11] were used for comparison purposes. Apart from the above, this work also reported the structural elucidation of cyclic dithiocarbonate derivative of ENR-50 obtained from the reaction between purified ENR-50 with neat carbon disulfide, CS_2_ [24]. The reaction is shown in Scheme 2. A complete ^1^H- and ^13^C-NMR chemical shifts assignments of the cyclic dithiocarbonate derivative of ENR-50 is given and the probable mechanism of formation was discussed.

**Table 1:** ^1^H, ^13^C-NMR chemical shifts and HMQC, HMBC and COSY spin coupling correlations of purified ENR-50.

^1^H chemical shift δ (ppm)			^13^C chemical shiftδ (ppm)		HMQC	HMBC Coupling correlation		COSY Coupling correlation	
Ref: 14–15	Ref: 11	This work	Ref: 11	This work	Triad assignment	Middle unit	Within same unit	Middle unit	Within same unit
						δ (ppm)	δ (ppm)	δ (ppm)	δ (ppm)
1.31	1.29	1.30	22.1	22.3	E^10^	29.7 (E^8^), 60.7 (E^6^), 64.5 (E^7^)	None	None	None
1.38	1.55	1.56	29.5	29.7	EE^8^C, E^8^	22.3 (E^10^), 60.7 (E^6^), 64.5 (E^7^)	24.7 (E^9^), 64.5 (E^7^)	None	1.68–1.70 (E^9^)
			33.0	33.1	CE^8^C, CE^8^E	22.3 (E^10^), 60.7 (E^6^), 64.5 (E^7^)	23.9 (C^4^), 125.0 (C^2^)	None	2.15–2.19 (C^4^)
			26.9	27.0	CE^9^C, EE^9^C	60.7 (E^6^), 64.5 (E^7^)	28.7 (C^3^), 134.7 (C^1^)	2.72 (E^7^)	2.15–2.19 (C^3^)
1.42	1.68	1.68–1.70	23.3	23.4	C^5^	32.0 (C^3^), 125.0 (C^2^), 134.7 (C^1^)	None	None	None
1.38			24.6	24.7	CE^9^E, E^9^	60.7 (E^6^), 64.5 (E^7^)	29.7 (E^8^), 60.7 (E^6^)	2.72 (E^7^)	1.56 (E^8^)
2.00	2.05	2.06	26.2	26.3	C^4^, EC^4^C	125.0 (C^2^), 134.7 (C^1^)	32.0 (C^3^), 134.7 (C^1^)	5.12–5.17 (C^2^)	2.06 (C^3^)
			32.0	32.0	C^3^, CC^3^E	23.4 (C^5^), 125.0 (C^2^), 134.7 (C^1^)	26.3 (C^4^), 125.0 (C^2^)	None	2.06 (C^4^)
1.96	2.15	2.15-2.19	23.7	23.9	CC^4^E, EC^4^E	125.0 (C^2^), 134.7 (C^1^)	33.1 (E^8^), 60.7 (E^6^)	5.12–5.17 (C^2^)	1.56 (E^8^)
			28.5	28.7	EC^3^C, EC^3^E	23.4 (C^5^), 125.0 (C^2^), 134.7 (C^1^)	27.0 (E^9^), 64.5 (E^7^)	None	1.56 (E^9^)
-	-	-	60.3	60.7	E^6^	-	**-**	-	-
2.51	2.70	2.72	64.0	64.5	E^7^	22.3 (E^10^), 24.7 (E^9^), 29.7 (E^8^), 60.7 (E^6^)	29.7 (E^8^), (None)	1.68-1.70 (E^9^)	None
5.20	5.10	5.12–5.17	125.0	125.0	C^2^	23.4 (C^5^), 26.3 (C^4^), 32.0 (C^3^), 134.7 (C^1^) (NOT DETECTED)	32.0 (C^3^)	2.06 (C^4^)	None
-	-	-	135.0	134.7	C^1^	-	-	-	-

## 2. Results and Discussion

### 2.1. Epoxidized Natural Rubber

2.1.1. ^1^H- and ^13^C-NMR Spectroscopy

**Figure 2:**
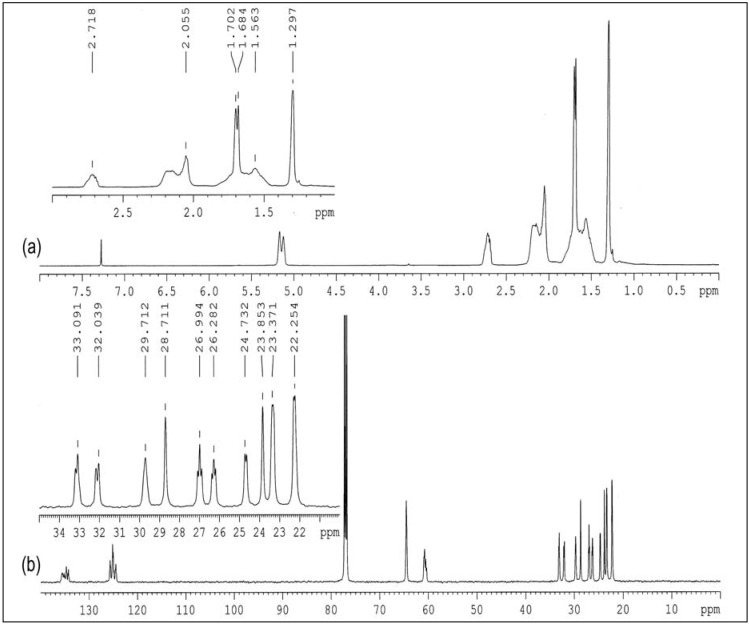
(**a**) ^1^H and (**b**) ^13^C-NMR spectra of purified ENR-50 (in CDCl_3_).

**Figure 3:**
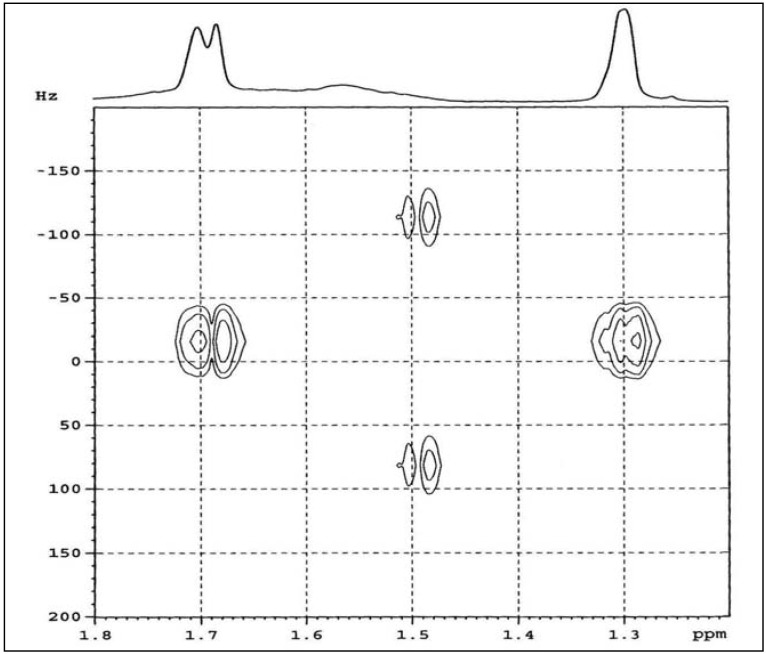
JRES spectra of purified ENR-50 (in CDCl_3_).

The ^1^H- and ^13^C-NMR spectra of purified ENR-50 are shown in Figure 2 and the respective chemical shifts are tabulated in Table 1. The respective observed chemical shifts were generally similar to those reported in previous works [11,14,15]. From Figure 2a, the ^1^H-NMR chemical shift at δ 1.30 ppm is assigned to the methyl protons of E^10^, at δ 2.72 ppm to the methine proton of E^7^ and at δ 5.12–5.17 ppm to the methine proton of C^2^. However, the chemical shift at δ 1.56 ppm overlapped with those at δ 1.68–1.70 ppm while the chemical shift at δ 2.15–2.19 ppm overlapped with those at δ 2.06 ppm. These overlapping signals may be due to the methylene protons of the ENR-50 chain. To confirm, a J-resolved spectroscopy (JRES) [25,26] experiment was conducted to specifically investigate the overlapping signals at δ 1.68–1.70 ppm region. Figure 3 shows the enlarged JRES spectra of δ 1.20–1.80 ppm region. The methyl of E^10^ is represented as a single contour at δ 1.30 ppm. The overlapping methylene signal in JRES was represented by a double contour located at δ 1.68 and 1.70 ppm, with coupling constant of 11 Hz. The multiple methylene protons signal is due to the different attachment modes of the epoxide to the isoprene which collectively form pairs of the enantiomers, stereoisomers and diastereoisomers that give rise to separate the resonances in the NMR spectrum [12].

The ^13^C-NMR spectrum of purified ENR-50 is shown in Figure 2b. The signals within δ 23.9 to 33.1 ppm represent the doublets of methylene carbons of C^3^, C^4^, E^8^ and E^9^. The methylene carbons of C^4^ and E^9^ are vicinal to the respective methine carbons of C^2^ and E^7^. While the methylene carbons of C^3^ and E^8^ are vicinal to the respective quaternary carbons of C^1^ and E^6^. The methine carbons of C^2^ and E^7^ has greater electron density than the quaternary carbons of C^1^ and E^6^ and therefore are more shielded causing the methylene carbons of C^4^ and E^9^ to be located in the upfield region compared to the methylene carbons of C^3^ and E^8^.

The ^13^C-NMR chemical shifts of the methylene carbons of C^4^ and E^9^ are also dependent on their vicinal neighboring units (*i.e.*, to the right (R)- or left (L)-hand side) in the triad. If E unit is on the R side of either C^4^ or E^9^, the chemical shift is more shielded and upfield than those with neighboring C unit on the same R side. Thus the chemical shifts of CC^4^E, EC^4^E, CE^9^E and E^9^ triads will be upfield compared to C^4^, EC^4^C, CE^9^C and EE^9^C triads. Similarly, when E is on the L side of C^3^ and E^8^ unit in the triad sequence, the chemical shifts of EC^3^C, EC^3^E, EE^8^C and E^8^ triads will be upfield compared to C when it is on the L side of C^3^ and E^8^ as in C^3^, CC^3^E, CE^8^C and CE^8^E triads. In the case of C^1^, C^2^, C^5^, E^6^, E^7^ and E^10^ triads, the ^13^C-NMR chemical shifts of the E series are located in the upfield region while of the C series are located in the downfield region. This is due to the double bond in the latter causes deshielding.

#### 2.1.2. HMQC

**Figure 4:**
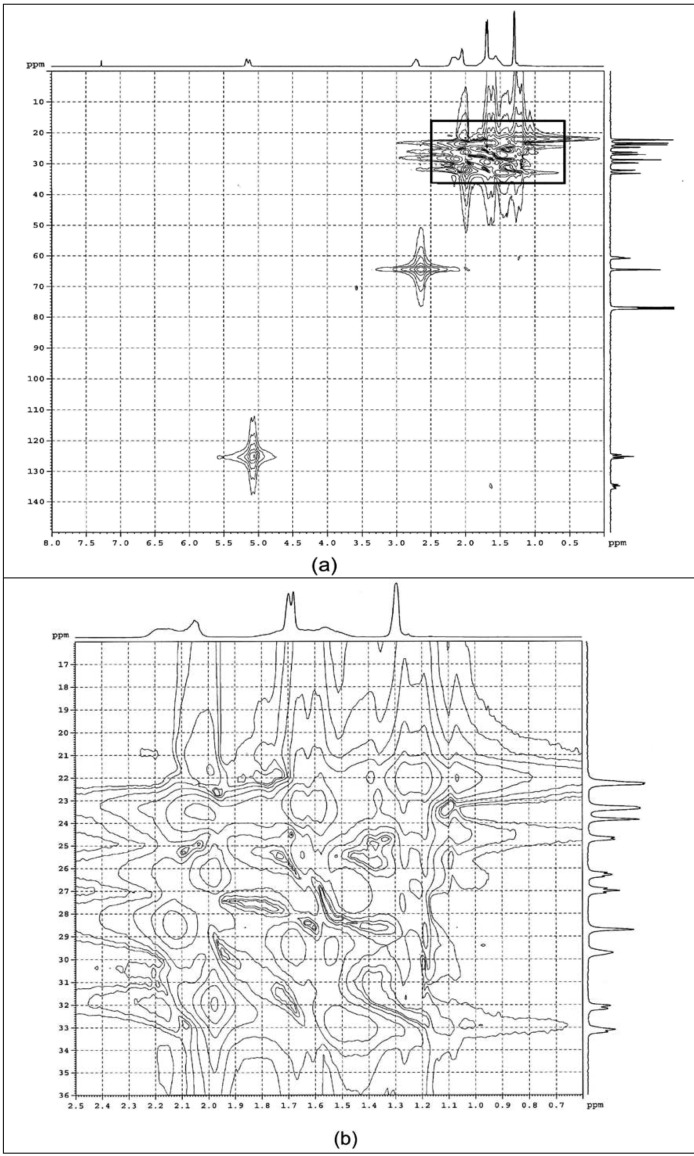
HMQC spectra of (**a**) purified ENR-50 and (**b**) enlargement of the boxed region in (**a**).

Figure 4 shows the HMQC spectra of purified ENR-50 and the identified triad assignments are tabulated in Table 1 (6th column). HMQC correlates the chemical shift of proton(s) directly bonded to carbon. The ^1^H-NMR chemical shift depends on the type of carbon it is attached to. The σ bond formed between the carbon and a proton produce shielding effect [27]. Thus depending on the number of C-H bond, protons attached to the primary carbon is shifted more upfield compared to the secondary and tertiary carbons.

The correlations of the methyls of E^10^ and C^5^ are located upfield than the methylenes of C^3^, C^4^, E^8^, E^9^ and methines of C^2^ and E^7^. The correlations arising from the methyls of E^10^ and C^5^ also depend on the types of vicinal quartenary carbon, *i.e.*, the epoxide (E^6^) or the double bond (C^1^). The methyl of E^10^ is vicinal to the quartenary carbon of E^6^ while the methyl of C^5^ is vicinal to quartenary carbon of C^1^. Thus the methyl of E^10^ is located upfield than that of methyl of C^5^. Similarly, the correlations of methines of E^7^ and C^2^ are influenced by the proximity of the epoxide or the double bond. The methine of E^7^ is shielded by electron density of epoxide while the methine of C^2^ is deshielded by the double bond. The methine of E^7^ is therefore more upfield than the methine of C^2^. In the case of methines of E^7^ and C^2^, their correlations are located downfield relative to the methylenes of C^3^, C^4^, E^8^ and E^9^. The deshielding effects of the double bond caused the correlations of methylenes of C^3^ and C^4^ to be located downfield than the respective methylenes of E^8^ and E^9^. Therefore C^3^ is also downfield than E^8^ while, C^4^ is downfield than E^9^.

For the EC^3^C and EC^3^E triads, these correlations are located downfield than CC^4^E and EC^4^E in the ^13^C-NMR spectrum. However, in the ^1^H-NMR spectrum, both appeared as overlapped peak. This is because the correlations of C^3^ and C^4^ superimpose those of CC^3^E and EC^4^C triads. Thus it can be similarly argued that the correlations of the triads CE^8^C and CE^8^E occur downfield than CE^9^C, and EE^9^C in the ^13^C-NMR spectrum while in the ^1^H-NMR spectrum, those of EE^8^C and CE^9^E superimpose those of E^8^ and E^9^.

#### 2.1.3. HMBC

**Figure 5:**
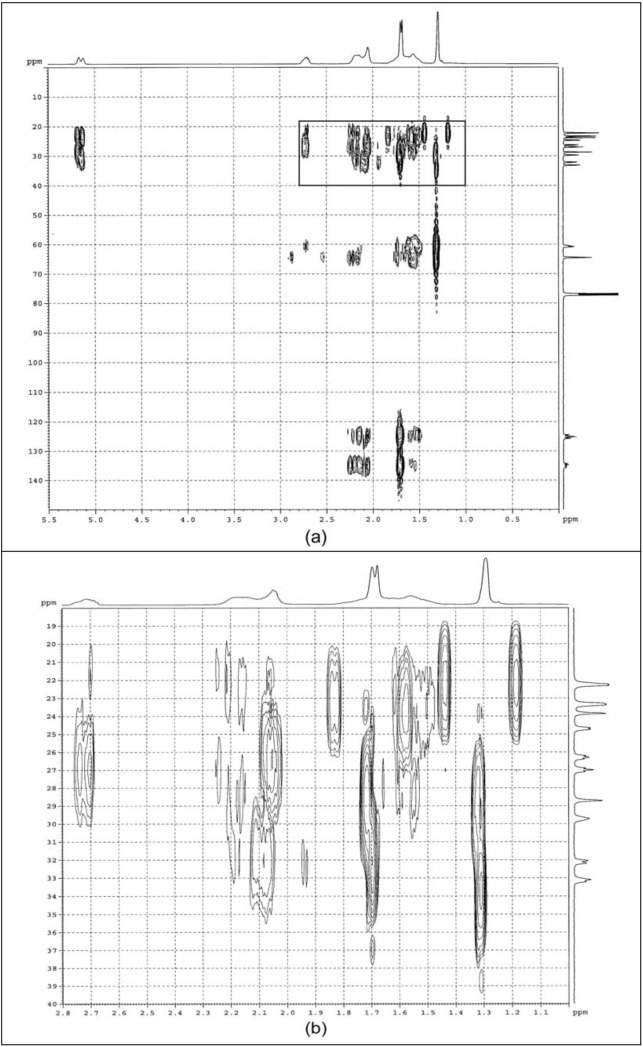
HMBC spectra of (**a**) purified ENR-50 and (**b**) enlargement of the boxed region in (**a**).

The HMQC results were further scrutinized using HMBC analysis. The HMBC correlation spectra of purified ENR-50 are shown in Figure 5. The ^1^H-^13^C signals correlations are tabulated in Table 1 (7th and 8th column). The overlapping proton signals at δ 1.56, 1.68–1.70, 2.06 and 2.15–2.19 ppm were individually assigned through coupling correlations. The coupling correlation between neighboring units classifies and arranges the methylene carbons in the correct triad sequence thus confirmed the previous HMQC assignments. The methyls of E^10^ and C^5^, and methines of E^7^ and C^2^ were affirmatively assigned based on the deduced correlations and consistent with the previous study [11].

#### 2.1.4. COSY

**Figure 6:**
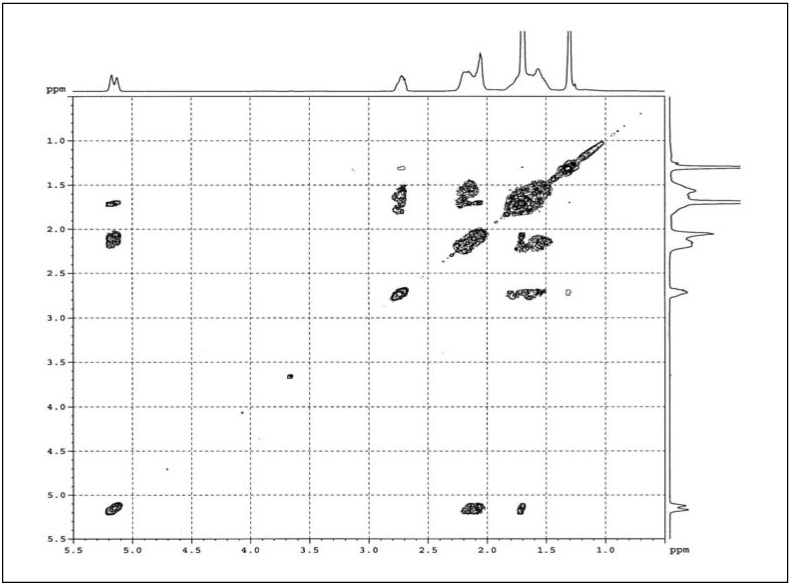
COSY spectra of purified ENR-50.

The COSY of purified ENR-50 is shown in Figure 6 and was conducted to evaluate the representations of ^1^H-NMR signals at δ 1.56, 1.68–1.70, 2.06 and 2.15–2.19 ppm. The spin coupling correlations of the signals and the assigned triad sequences are as tabulated in column 9th and 10th in Table 1.

The signal at δ 1.56 ppm correlates with the signals at δ 2.72, 2.15–2.19 and 1.68–1.70 ppm. The signal of E^7^ methine proton at δ 2.72 ppm correlates to the methylene protons of the middle unit of the triad sequences of CE^9^C and EE^9^C. The signal representing the methylene protons of CC^4^E and EC^4^E as well as EC^3^C and EC^3^E at δ 2.15–2.19 ppm correlates to the methylene protons of isoprene CE^8^C, CE^8^E, CE^9^C and EE^9^C. While the signal assigned to the methyl protons of C^5^, methylene protons of CE^9^E and E^9^ at δ 1.68–1.70 ppm correlates to methylene protons of the epoxidized isoprene of EE^8^C and E^8^. The methyl proton of E^10^ at δ 1.30 ppm does not show any correlation as it is attached to the quartenary carbon, E^6^.

The signal that represents the methylene protons of the middle unit of C^4^ and EC^4^C as well as C^3^ and CC^3^E at δ 2.06 ppm correlates with the signals at δ 2.06 and 5.12–5.17 ppm. All these methylene protons are correlated to the methylene protons of isoprene with the same unit of triad at δ 2.06 ppm. However, for the triads C^4^ and EC^4^C, the correlation is to the methine proton of isoprene C^2^ at δ 5.12–5.17 ppm.

The methylene protons of CC^4^E and EC^4^E at δ 2.15–2.19 ppm were correlated with the signals at δ 5.12–5.17 and 1.56 ppm. These are due to the methine proton of C^2^ (δ 5.12–5.17 ppm) and the neighbouring methylene protons of epoxidized isoprene (δ 1.56 ppm) within the same unit. The methylene protons of EC^3^C and EC^3^E at δ 2.15–2.19 ppm are also correlated to the neighbouring methylene protons of epoxidized isoprene (δ 1.56 ppm) within the same unit of triad sequence. 

Based on the COSY of purified ENR-50, the signal at δ 1.56 ppm represents the methylene protons of CE^9^C, EE^9^C, E^8^, EE^8^C, CE^8^C and CE^8^E. The signal at δ 1.68–1.70 ppm represents the methyl protons of C^5^ and the methylene protons of CE^9^E and E^9^. The signal at δ 2.06 ppm arises from the methylene protons of C^4^, EC^4^C, C^3^ and CC^3^E, while the signal at δ 2.15–2.19 ppm represents the methylene protons of CC^4^E, EC^4^E, EC^3^C and EC^3^E. These conformed to the HMQC results above.

### 2.2. Cyclic Dithiocarbonate Derivative of ENR-50

The reaction of CS_2_ with purified ENR-50 afforded the five-membered ring dithiocarbonate derivative of ENR-50 as shown in Scheme 2. The formation of cyclic dithiocarbonate is generally a Diels-Alder type of reaction [24]. In this work, a very dilute solution of purified ENR-50 was used with the aim to stretch the random coil conformation of ENR-50 polymer chains in order to expose the reactive epoxidized isoprene unit using dimethylaminopyridine (DMAP) as the catalyst. The C–S moiety of CS_2_ is inserted into the epoxidized isoprene of the ENR-50. The reaction probably occurs via the S_N_2 mechanism that involved the epoxide ring opening. 

#### 2.2.1. FTIR Spectroscopy

**Figure 7:**
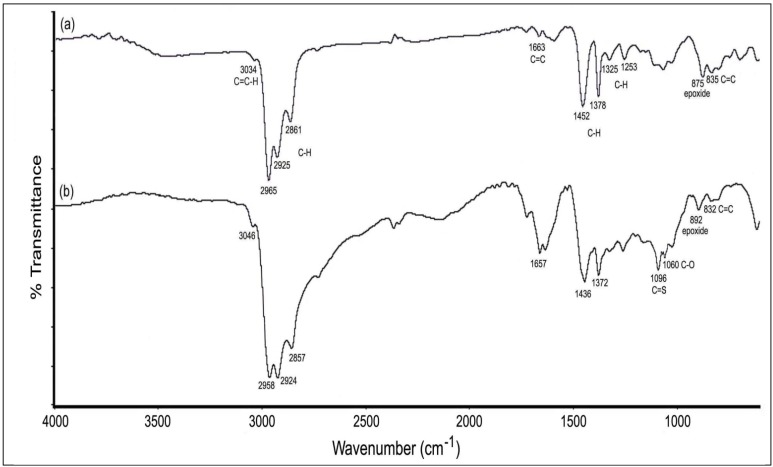
FTIR spectra of (**a**) purified ENR-50 and (**b**) cyclic dithiocarbonate derivative of ENR-50.

Comparison of the FTIR spectrum of purified ENR-50 (Figure 7a) with the FTIR spectrum of the reaction product (Figure 7b) revealed two new bands at 1096 and 1060 cm^−1^ that are assigned to C=S and C-O functionalities, respectively [28,29]. The latter (υ_C-O_) is located at lower wavenumber than the υ_C-O-C_ for cyclic ether at 1150–1070 cm^−1^. The weak C-S stretching vibration in the range of 700–590 cm^−1^ indicates bonding between sulfur from CS_2_ and the carbon E^7^ [29]. The band attributed to the epoxide is shifted to higher wavenumber compared to purified ENR-50, *i.e.*, from 875 to 892 cm^−1^. Other typical bands of C=C and C–H are also slightly shifted to a low wavenumber region. This is probably due to the intermittent presence of cyclic dithiocarbonate within the ENR-50 chains.

Comparison of the spectra of Figure 7a,b also indicates that there is a reduction in the epoxide band intensity after the reaction. The extent of dithiocarbonation reaction was estimated using semi-quantitative FTIR approach. This was based on the band area of the methyl, C=S and the epoxide functionalities before and after dithiocarbonation reaction. The band due to methyl at 1378–1372 cm^−1^ was used as the internal standard in this work [8]. Based on the FTIR approach, the percentage of epoxidation in the purified ENR-50 is ~50.7%. It was also found that after the dithiocarbonation reaction, 47.9% of the epoxide has reacted with CS_2_ while 52.1% were unreacted.

#### 2.2.2. ^1^H and ^13^C-NMR Spectroscopy

**Figure 8:**
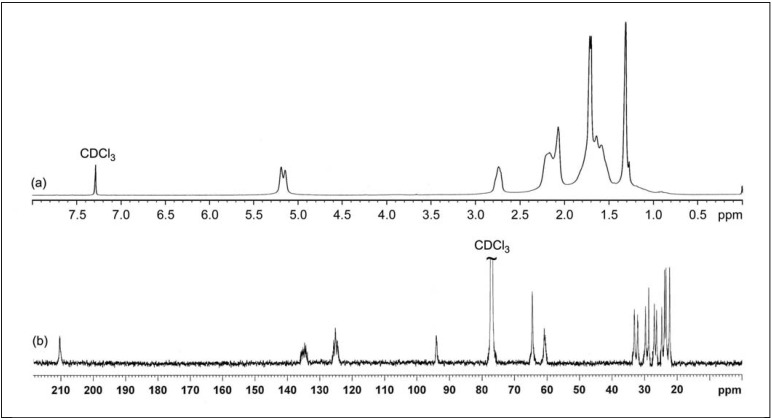
(**a**) ^1^H and (**b**) ^13^C-NMR spectra of cyclic dithiocarbonate derivative of ENR-50 (in CDCl_3_).

The ^1^H and ^13^C-NMR spectra of cyclic dithiocarbonate derivative of ENR-50 are shown in Figure 8. The chemical shifts of the cyclic dithiocarbonate derivative of ENR-50 as well as the triad and carbon assignments are given in Table 2. From Figure 8a, with the exception for the splitting of peak at δ 1.58–1.63 ppm, the ^1^H-NMR spectrum shows almost similar features compared to purified ENR-50 (Figure 2a). As discussed earlier, the split peak above represents the methylene protons of EE^8^C, E^8^, CE^8^C, CE^8^E, CE^9^C and EE^9^C triads. The formation of cyclic five-membered dithiocarbonate within the ENR chain involves the E^6^ and E^7^ carbons as illustrated in Scheme 2. This, in turn, has affected the environment of E^8^ and E^9^ carbons. Therefore the split peaks observed at δ 1.58–1.63 ppm may be attributed to the methylene protons attached to the five-membered (cylic dithiocarbonate) and three-membered (oxirane) rings, respectively. The methylene protons attached to the five-membered ring are slightly downfield at δ 1.63 ppm compared to those attached to the three-membered ring (at δ 1.58 ppm).

**Table 2:** ^1^H and ^13^C-NMR chemical shifts and triad assignments for purified ENR-50 and its cyclic dithiocarbonate derivative.

Chemical shift δ (ppm)				Triad assignment
^1^H		^13^C		
ENR-50 *	Cyclic dithiocarbonate derivative	ENR-50 *	Cyclic dithiocarbonate derivative	
1.30	1.31	22.3	22.4	E^10^
1.56	1.58–1.63	29.7	29.8	EE^8^C, E^8^
		33.1	33.1	CE^8^C, CE^8^E
		27.0	27.0	CE^9^C, EE^9^C
1.68–1.70	1.70–1.71	23.4	23.5	C^5^
		24.7	24.8	CE^9^E, E^9^
2.06	2.08	26.3	26.4	C^4^, EC^4^C
		32.0	32.2	C^3^, CC^3^E
2.15–2.19	2.12–2.19	23.9	24.0	CC^4^E, EC^4^E
		28.7	28.7	EC^3^C, EC^3^E
-	-	60.7	61.0	E^6^
2.72	2.73	64.5	64.8	E^7^, E^7^of five-membered ring
-	-	-	94.0	E^6^ of five-membered ring
5.12–5.17	5.15–5.19	125.0	125.1	C^2^
-	-	134.7	134.9	C^1^
-	-	-	210.5	CS_2_ carbon of five-membered ring

* from Table 1.

These methylene protons shows equal proton integrals compared to purified ENR-50. This is confirmed by the previous FTIR results where, approximately 1:1 ratio of reacted and unreacted epoxide is observed after the dithiocarbonation reaction. The formation of cyclic dithiocarbonate within the ENR chain however, does not produce any new peaks or change in the chemical shifts. Therefore it is expected that the ENR-50 polymer chains conformation was maintained upon dithiocarbonation.

The formation of cyclic dithiocarbonate also gives two new ^13^C-NMR peaks at δ 210.5 and 94.0 ppm, respectively [24]. The peak at δ 210.5 ppm represents the carbon of >C=S of the five-membered ring and is located at the most downfield region as this carbon is simultaneously bonded to the electronegative oxygen and sulfur. The carbon E^6^ of the five-membered ring is located slightly downfield at δ 94.0 ppm because it is vicinally bonded to the carbon of >C=S. The chemical shift for carbon E^7^ of the five-membered ring and the carbons of E^6^ and E^7^ of the three-membered ring (oxirane) however, remains unchanged, *i.e.*, similar to the purified ENR-50. The main difference between the cyclic dithiocarbonated ENR reported in this work and the previously reported cyclic carbonated NR [30] is the position of S atom in the former is replaced by O atom in the latter. This gives rise to the different chemical shifts due to the different in electronegativity of S and O atoms. In the cyclic carbonated NR, the ^13^C-NMR chemical shift of quartenary carbon of >C=O is at δ 151.0 pm, the tertiary carbon of >C(H)–O– is at δ 75.0 ppm, and the quartenary carbon of ≡C–O– is at δ 74.0 ppm, respectively [30].

The ^13^C-NMR quantitative analysis was carried out to evaluate the epoxidation level in the purified ENR-50 as well as its cyclic dithiocarbonate derivative. The epoxidation level in purified ENR-50 was found to be ~51.4%. In the cyclic dithiocarbonate derivative, 52.0% of the epoxide ring is unreacted while 48.0% has reacted with CS_2_. These results are consistent with the FTIR results discussed previously. 

**Figure 9:**
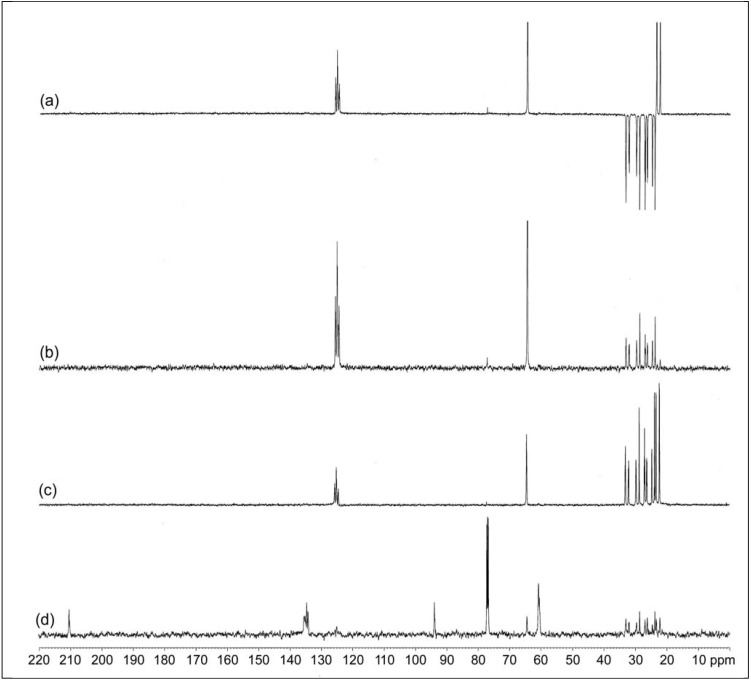
(**a**) DEPT-135; (**b**) DEPT-90; (**c**) DEPT-45 and (**d**) DEPT quartenary spectra of cyclic dithiocarbonate derivative of ENR-50 (in CDCl_3_).

Figure 9 shows the various DEPT spectra for cyclic dithiocarbonate derivative of ENR-50. In DEPT-135, the signals of the methylene carbons are shown downward (negative) while the methyl and methine carbons are shown as the upward peaks (positive) [31]. The spectrum for DEPT-90 shows only the methine carbons [31]. However, spectrum for DEPT-45 shows the upward peaks for methyl, methylene and methine carbons. The peak at δ 64.8 ppm appears as upward signal in DEPT-135, DEPT-90 and DEPT-45 proved its assignment as a methine carbon for E^7^ of the three-membered ring (oxirane) as well as carbon E^7^ of the five-membered ring. DEPT quartenary only shows quartenary carbon as upward peak in the spectrum. The real signals of the quartenary carbons in this spectrum are at δ 60.1, 94.0, 134.9 and 210.5 ppm. While the rest of the peaks in this spectrum are not reliable because the signal to noise ratio is less than 5 and those peaks are proven previously as methyl, methylene and methine in DEPT-135, -90 and -45 except for the CDCl_3_ peak which appear at 77.0 ppm. Thus the peaks at δ 94.0 ppm and 210.5 ppm are assigned for E^6^ and CS_2_ carbon of five-membered ring, respectively.

#### 2.2.3. Proposed Mechanism for the Formation of Cyclic Dithiocarbonate

**Scheme 3:**
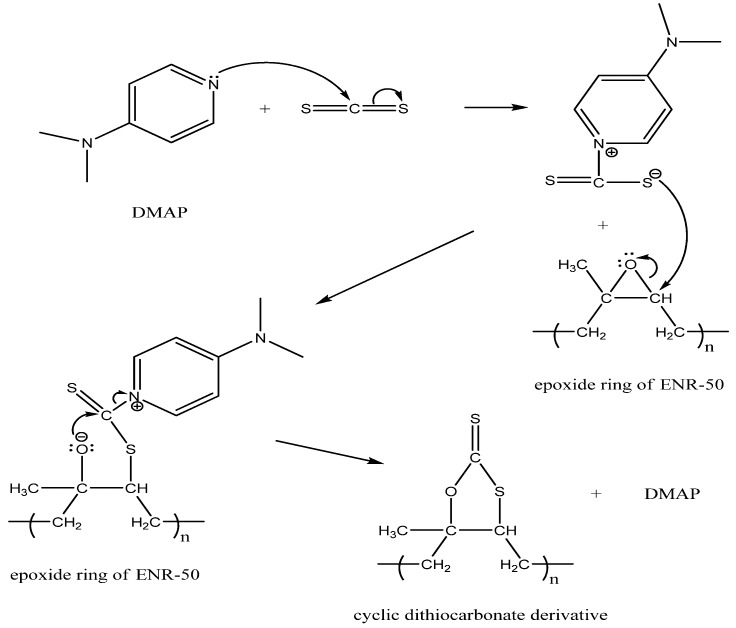
Proposed mechanism for the formation of cyclic dithiocarbonate derivative of ENR-50 via oxirane ring opening.

A proposed mechanism for the formation of cyclic dithiocarbonate from the reaction of ENR-50 with CS_2_ is shown in Scheme 3. DMAP catalyst is well known as a strong nucleophile. The lone pair electrons of DMAP attack the carbon of CS_2_ and form a [S_2_C-DMAP] complex. The complex provides the condition for overall reaction to proceed via a S_N_2 mechanism [32]. In this condition, the sulfur-centered nucleophile attacks the less substituted and less sterically hindered carbon E^7^ and ring opened the oxirane. The bond between oxygen and carbon E^7^ is severed and produced oxygen-centered nucleophile. This nucleophile attacks the CS carbon of [ENR-S(S)C-DMAP] intermediate with simultaneous detachment of a DMAP molecule and therefore affecting a ring closure via formation of O–C bond. The ring closure constitutes formation of cyclic dithiocarbonate derivative of ENR-50. 

## 3. Experimental

### 3.1. Materials

Carbon disulfide 99.7%, CS_2_ (BDH Chemicals, Poole, UK), 4-dimethylaminopyridine 98%, DMAP and deuterated chloroform, CDCl_3_ (Fluka Chemicals, Buchs, Switzerland) and *n*-hexane (Systerm, Selangor, Malaysia) were all obtained commercially and used without further purification unless otherwise stated. Epoxidized natural rubber with 50% epoxidation (ENR-50) was purchased from the Rubber Research Institute (Kuala Lumpur, Malaysia). 

### 3.2. Preparative Procedure

#### 3.2.1. Purification of ENR-50 [19]

ENR-50 (about 20.00 g) was swelled in chloroform (400 mL) and stirred for 24 h at room temperature. The solution was then filtered through cotton gauze to separate the gel (high molecular ENR-50) from the extract (low molecular weight ENR-50). The later was precipitated in *n*-hexane and stirred using a glass rod. The white precipitate stuck to the glass rod was transferred to a Petri dish and was dried in a vacuum oven at 50 °C for two days. Mass of purified ENR-50 sample was recorded daily until a constant weight was achieved. 

#### 3.2.2. Reaction of Purified ENR-50 with CS_2_ [24]

Purified ENR-50 (about 20 mg, 1.31 × 10^−4^ mol) was swelled in CS_2_ (40 mL, 6.62 × 10^−4^ mol) under constant stirring. DMAP catalyst (about 16.44 mg, 1.32 × 10^−4^ mol) was added to the stirring solution. The mixture was then refluxed for 3 h and then left to cool to room temperature. The organic layer was washed with water (150 mL) to remove the catalyst. This procedure was repeated until the washing attained a neutral pH. The organic layer was then cast onto Teflon dishes before drying in a vacuum oven at 50 °C for 24 h.

### 3.3. Measurements and Characterization Techniques

FTIR spectra were recorded on a Perkin-Elmer 2000-FTIR using single beam transmittance onto a film of sample on ZnSe window in the range of 4,000–600 cm^−1^. The FTIR samples were prepared by swelling sample (100.00 mg) with chloroform (5 mL). The sample solution was cast onto a Teflon mould and air dried. The thin film was dried in a vacuum oven at 50 °C for 1 h and was later transferred onto ZnSe window. 1D and 2D NMR spectra were obtained using a Bruker Avance 500 MHz instrument in CDCl_3_ at 25 °C. A 10.00 mg sample was used for ^1^H and J-Resolved Spectroscopy (JRES) while 50.00 mg were used for the ^13^C-NMR, distortion enhancement by polarization transfer (DEPT), heteronuclear multiple quantum coherence (HMQC), heteronuclear multiple bond coherence (HMBC) and correlation spectroscopy (COSY) analyses. The respective range of the spectra and number of scan for ^1^H and for ^13^C-NMR measurements were 15–0 ppm with 16 scans and 200–0 ppm with 15,000 scans, respectively. For quantitative ^13^C-NMR measurements, 1,000 scans were applied with 60 seconds relaxation delay. The number of scans applied for JRES, DEPT, HMQC, HMBC and COSY were 8, 2000, 128, 256 and 40 scans, respectively.

### 3.4. Theoretical Treatments

The FTIR semi-quantitative treatments were based on the band area of methyl, epoxide and C=S functional groups. The percentage of epoxide in purified ENR-50 and the percentage dithiocarbonation of the epoxide were determined using Equations (1) and (2), respectively. In Equations (1) and (2), N_methyl_ is the normalized band area of the methyl signal, A _epoxide sample_ is the band area of epoxide functional group stretching peak for the sample, A _C=S sample_ is the band area of C=S functional group stretching peak for the sample, and A _epoxide ENR-50_ is the band area of respective functional group stretching peak in purified ENR-50: 


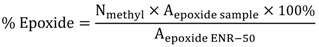
(1)


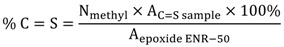
(2)

The ^13^C-NMR quantitative treatments were also used to determine the percentage of epoxidation in the purified ENR-50 and its five-membered ring cyclic dithiocarbonate derivative using Equations (3) and (4) below. In both equations, I_C1_ is the integral of C^1^ at δ 134.9 ppm, I_E6 epoxide_ is the integral of E^6^ at δ 61.0 ppm, and I_E6 cyclic_ is the integral of E^6^ at δ 94.0 ppm:


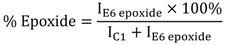
(3)


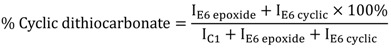
(4)

## 4. Conclusions

The overlapping ^1^H-NMR signals of purified ENR-50 at δ 1.56, 1.68–1.70, 2.06, 2.15–2.17 ppm was successfully assigned to their respective triad sequences using the 2D NMR; HMQC, HMBC and COSY techniques. The signal at δ 1.56 ppm was assigned to methylene protons of epoxidized isoprene, δ 1.68–1.70 ppm to both methyl protons of isoprene and methylene protons of epoxidized isoprene. The signals at δ 2.06 and 2.17–2.19 ppm were both assigned to the methylene protons of isoprene. The ^13^C-NMR chemical shifts were similar to those previously reported. However, the chemical shifts of methylene carbons were dependent on the types of vicinal neighbouring units in the triad sequence. The reaction of purified ENR-50 with CS_2_ formed the cyclic dithiocarbonate derivative of ENR-50 involving ring opening of the oxirane and the insertation of the C-S moiety at the oxygen attached to the quartenary carbon and methine carbon of epoxidized isoprene unit respectively. FTIR analysis showed that the C=S and C–O absorption bands appeared at 1096 and 1060 cm^−1^, respectively. ^1^H-NMR spectrum of cyclic dithiocarbonate derivative of ENR-50 displays splitting at δ 1.58–1.63 ppm and was assigned to the methylene protons of the respective epoxide and five-membered dithiocarbonate rings. Meanwhile, ^13^C-NMR showed two new carbon peaks assigned to the >C=S and the quartenary carbon of five-membered cyclic dithiocarbonate ring. The FTIR semi-quantitative and ^13^C-NMR quantitative calculations revealed that approximately half of the epoxide units in the ENR-50 were converted to the dithiocarbonate derivative.

## References

[1] Baker C.S.L., Gelling I.R., Gelling I.R. (1987). Epoxidized Natural Rubber. Development of Rubber Technology.

[2] Gelling I.R. (1985). Modification of Natural Rubber with Peracetic Acid. Rubber Chem. Technol..

[3] Jeerupun J., Wootthikanokkhan J., Phinyocheep P. (2004). Effects of Epoxidation Content of ENR on Morphology and Mechanical Properties of Natural Rubber Blended PVC. Macromol. Symp..

[4] Lee S.Y., Hassan A., Tan I.K.P., Terakawa K., Ichikawa N., Gan S.N. (2010). Reaction of Palm Oil Based mcl-PHAs with Epoxidized Natural Rubber. J. Appl. Polym. Sci..

[5] Gelling I.R. (1991). Epoxidised Natural Rubber. J. Nat. Rubb. Res..

[6] Burfield D.R., Lim K.L., Law K.S., Ng S. (1984). Analysis of Epoxidized Natural Rubber: A Comparative Study of D.S.C., N.M.R., Elemental Analysis and Direct Titration Methods. Polymer.

[7] Gelling I.R., Salamone J.C. (1996). Epoxidized Natural Rubber. Polymeric Materials Encyclopedia.

[8] Bhattacharjee S., Bhowmick A.K., Avasthi B.N. (1993). Hydrogenation of Epoxidized Natural Rubber in the Presence of Palladium Acetate Catalyst. Polymer.

[9] Gan S.N., Ziana A.H. (1997). Partial Conversion of Epoxide Groups to Diols in Epoxidized Natural Rubber. Polymer.

[10] Derouet D., Brosse J.C., Challioui A. (2001). Alcoholysis of Epoxidized Polyisoprene by Direct Opening of Oxirane Rings with Alcohol Derivatives. 2. Study on Epoxidized 1,4-Polyisoprene. Eur. Polym. J..

[11] Saito T., Klinklai W., Kawahara S. (2007). Characterization of Epoxidized Natural Rubber by 2D NMR Spectroscopy. Polymer.

[12] Bradbury J.H., Perera M.C.S. (1985). Epoxidation of Natural Rubber Studied by NMR Spectroscopy. J. Appl. Polym. Sci..

[13] Thames S.F., Gupta S. (1997). Synthesis and Characterization of Pendent Hydroxy Fluoroesters of Secondary High Molecular Weight Guayule Rubber. J. Appl. Polym. Sci..

[14] Furst A., Pretsch E. (1990). A Computer Program for the Prediction of ^13^C-NMR Chemical Shifts of Organic Compounds. Anal. Chim. Acta.

[15] Pretsch E., Furst A., Badertscher M., Burgin R. (1992). C13Shift: A Computer Program for the Prediction of ^13^C-NMR Spectra Based on an Open Set of Additivity Rules. J. Chem. Inf. Comput. Sci..

[16] Mohamad Z., Ismail H., Chantara Thevy R. (2006). Characterization of Epoxidized Natural Rubber/Ethylene Vinyl Acetate (ENR-50/EVA) Blend: Effect of Blend Ratio. J. Appl. Polym. Sci..

[17] Noriman N.Z., Ismail H., Rashid A.A. (2010). Characterization of Styrene Butadiene Rubber/Recycled Acrylonitrile-butadiene Rubber (SBR/NBRr) Blends: The Effects of Epoxidized Natural Rubber (ENR-50) As a Compatibilizer. Polym. Test..

[18] Abu Bakar M., Ismail J., Teoh C.H., Tan W.L., Abu Bakar N.H.H. (2008). Modified Natural Rubber Induced Aqueous to Toluene Phase Transfer of Gold and Platinum Colloids. J. Nanomater..

[19] Lee H.K., Ismail J., Kammer H.W., Bakar M.A. (2005). Melt Reaction in Blends of Poly(3-Hydroxybutyrate) (PHB) and Epoxidized Natural Rubber (ENR-50). J. Appl. Polym. Sci..

[20] Han C.C., Ismail J., Kammer H.W. (2004). Melt Reaction in Blends of Poly(3-hydroxybutyrate-co-3-hydroxyvalerate) and Epoxidized Natural Rubber. Polym. Degrad. Stab..

[21] Mishra J.K., Chang Y.W., Kim D.K. (2007). Green Thermoplastic Elastomer Based on Polycaprolactone/Epoxidized Natural Rubber Blend as a Heat Shrinkable Materials. Mater. Lett..

[22] Nghia P.T., Siripitakchai N., Klinklai W., Saito T., Yamamoto Y., Kawahara S. (2008). Compatibility of Liquid Deproteinized Natural Rubber Having Epoxy Group (LEDPNR)/Poly (L-Lactide) Blend. J. Appl. Polym. Sci..

[23] Nghia P.T., Onoe H., Yamamoto Y., Kawahara S. (2008). Hydrogenation of Natural Rubber Having Epoxy Group. Colloid Polym. Sci..

[24] Halimehjani A.Z., Ebrahimi F., Azizi N., Saidi M.R. (2009). A Simple and Novel Eco-Friendly Process for the Synthesis of Cyclic Dithiocarbonates from Epoxides and Carbon Disulfide in Water. J. Heterocycl. Chem..

[25] Giraudeau P., Akoka S. (2008). Resolution and Sensitivity Aspects of Ultrafast *J*-resolved 2D NMR Spectra. J. Magn. Reson..

[26] Viant M.R. (2003). Improved Methods for the Acquisition and Interpretation of NMR Metabolomic Data. Biochem. Biophys. Res. Commun..

[27] Macomber R.S. (1998). A Complete Introduction to Modern NMR Spectroscopy.

[28] Shamsuzzaman, Salim A. (1997). Synthesis of 1,3-Oxathiolane-2-Thiones by the Reaction of Steroidal Oxiranes with Carbon Disulfide. Tetrahedron Lett..

[29] Conley R.T. (1972). Infrared Spectroscopy.

[30] Kawahara S., Saito T. (2006). Preparation of Carbonated Natural Rubber. J. Polym. Sci. A Polym. Chem..

[31] Kawahara S., Chaikumpollert O., Sakurai S., Yamamoto Y., Akabori K. (2009). Crosslinking Junctions of Vulcanized Natural Rubber Analyzed by Solid-state NMR Spectroscopy Equipped with Field-gradient-magic Angle Spin Probe. Polymer.

[32] Bruice P.Y. (2001). Organic Chemistry.

